# Emergency Physician Use of the Alberta Netcare Portal, a Province-Wide Interoperable Electronic Health Record: Multi-Method Observational Study

**DOI:** 10.2196/10184

**Published:** 2018-09-25

**Authors:** Timothy AD Graham, Mark Ballermann, Eddy Lang, Michael J Bullard, Denise Parsons, Gabriella Mercuur, Pat San Agustin, Samina Ali

**Affiliations:** 1 Alberta Health Services Edmonton, AB Canada; 2 Department of Emergency Medicine University of Alberta Edmonton, AB Canada; 3 Department of Critical Care Medicine University of Alberta Edmonton, AB Canada; 4 Department of Emergency Medicine University of Calgary Calgary, AB Canada; 5 Department of Paediatrics University of Alberta Edmonton, AB Canada

**Keywords:** ambulatory care facilities, cross-sectional studies, electronic health records, utilization, hospital emergency service, statistics and numerical data, health information exchange, humans, information dissemination, medical record linkage, program evaluation

## Abstract

**Background:**

The adoption and use of an electronic health record (EHR) can facilitate real-time access to key health information and support improved outcomes. Many Canadian provinces use interoperable EHRs (iEHRs) to facilitate health information exchange, but the clinical use and utility of iEHRs has not been well described.

**Objective:**

The aim of this study was to describe the use of a provincial iEHR known as the Alberta Netcare Portal (ANP) in 4 urban Alberta emergency departments. The secondary objectives were to characterize the time spent using the respective electronic tools and identify the aspects that were perceived as most useful by emergency department physicians.

**Methods:**

In this study, we have included 4 emergency departments, 2 using paper-based ordering (University of Alberta Hospital [UAH] and Grey Nuns Community Hospital [GNCH]) and 2 using a commercial vendor clinical information system (Peter Lougheed Centre [PLC] and Foothills Medical Centre [FMC]). Structured clinical observations of ANP use and system audit logs analysis were compared at the 4 sites from October 2014 to March 2016.

**Results:**

Observers followed 142 physicians for a total of 566 hours over 376 occasions. The median percentage of observed time spent using ANP was 8.5% at UAH (interquartile range, IQR, 3.7%-13.3%), 4.4% at GNCH (IQR 2.4%-4.4%), 4.6% at FMC (IQR 2.4%-7.6%), and 5.1% at PLC (IQR 3.0%-7.7%). By combining administrative and access audit data, the median number of ANP screens (ie, results and reports displayed on a screen) accessed per patient visit were 20 at UAH (IQR 6-67), 9 at GNCH (IQR 4-29), 7 at FMC (IQR 2-18), and 5 at PLC (IQR 2-14). When compared with the structured clinical observations, the statistical analysis of screen access data showed that ANP was used more at UAH than the other sites.

**Conclusions:**

This study shows that the iEHR is well utilized at the 4 sites studied, and the usage patterns implied clinical value. Use of the ANP was highest in a paper-based academic center and lower in the centers using a commercial emergency department clinical information system. More study about the clinical impacts of using iEHRs in the Canadian context including longer term impacts on quality of practice and safety are required.

## Introduction

### Background

The adoption and use of an electronic health record (EHR) can facilitate real-time access to key health information and support improved outcomes [1-9]. Canadian hospitals are still primarily paper based, with only 5.2% of hospitals progressing to stage five or higher on the 7-point Electronic Medical Record Adoption Scale [10]. Although most hospital-based care is still documented on paper, there has been systematic investment and adoption of digital ancillary systems such as laboratory, pharmacy, and diagnostic imaging, sponsored by Canada Health Infoway (*Infoway*). Infoway is an independent, not-for-profit organization funded by the federal government, which works with the provinces and territories to cofund digital health projects throughout Canada.

One of the Canadian strategies for digitizing health care has been the promotion of the interoperable EHR (iEHR), which is equivalent to the health information exchange concept used in other jurisdictions [11,12]. iEHRs are being developed by each province as a part of the wider Canadian initiative to connect health care nationally. iEHRs are intended to be a longitudinal summary of key health events and act as a complement to point-of-service systems such as the electronic *medical* record (EMR) [13]. iEHRs have been shown to reduce duplicative laboratory and radiology testing, emergency department costs, and hospital admissions, with most clinicians also attributing positive changes in care coordination, communication, and knowledge about patients [14]. In contrast to the iEHR, in Canada, EMRs tend to be facility centric and refer to an ambulatory care record primarily linked to one single-care environment (such as a particular physician’s office) or a hospital-based clinical information system.

In Canada, 12 of the 13 provincial and territorial governments have implemented iEHRs, but their maturity is quite variable. To date, only 5 of the 12 have made the 4 planned clinical components available, including diagnostic images, laboratory test results, dispensed drugs, clinical reports, and immunizations [15].

Alberta (population 4.2 million) was the first Canadian province to develop a provincial read-only iEHR known as the Alberta Netcare Portal (ANP). The ANP iEHR provides read-only access to province-wide laboratory results, diagnostic imaging (reports and images), electrocardiograms, textual reports (dictated discharge summaries, operative reports, some consultations, etc), scanned reports (including the paper emergency department charts from the Edmonton Zone), dispensed drug information via the provincial Pharmaceutical Information Network, and contains data from more clinical domains than any other province [16,17]. ANP has become an important tool for continuity of care, and although inpatient care is provided on paper charts, the information in the ANP has almost entirely supplanted the need to refer to paper charts in the emergency department. This trend was seen early in the deployment of the ANP [18].

The ANP was introduced in 2004 in 13 hospitals, 22 public health centers, 9 mental health clinics, and a small number of private physician offices within the Edmonton Zone of Alberta Health Services (formerly known as Capital Health Authority) and was rapidly adopted by providers in diverse care environments [17,18].

In 2008, there was a substantial reorganization of health care in Alberta, and multiple health regions were amalgamated into the first Canadian provincial Health Authority called Alberta Health Services (AHS). Currently, AHS comprises 5 functional and regional zones, each with varying degrees of health care digitization. In acute care facilities, including emergency departments, the vast majority of the Edmonton Zone uses paper for documentation and order entry, with digital radiology as well as electronic patient tracking and laboratory results delivery. In contrast, the Calgary Zone uses an emergency department and inpatient clinical information system from a commercial vendor (Allscripts), with virtually 100% computerized provider order entry. The ANP is available as a tab and launched from within the Allscripts interface.

Currently, ANP is used in a read-only fashion in 111 hospitals and more than 650 facilities affiliated within AHS, as well as family practice clinics, pharmacies, and other health care entities in the province. In 2018, over 51,000 physicians, nurses, pharmacists, and other Alberta health care providers had role-based ANP access, with more than 1.9 million patient records accessed monthly [19]. The ANP’s evolution from a regional to a province-wide system and the usage patterns and contrasting information systems between zones in AHS presented a natural experiment and an excellent opportunity to study the ANP’s utility and usage in 4 busy urban emergency departments.

### Objective

The primary objective of this study was to compare the use of an iEHR (health information exchange-type) called ANP and other ancillary electronic systems between emergency department physicians practicing primarily in paper-based emergency departments versus clinical information system-based emergency departments. The secondary objectives were to characterize the time spent using the respective electronic tools and identify the aspects that were perceived as most useful by emergency department physicians.

## Methods

### Setting and Participants

We included 4 emergency departments in the study, 2 in the Edmonton Zone (University of Alberta Hospital [UAH] and Grey Nuns Community Hospital [GNCH]) and 2 in the Calgary Zone (Peter Lougheed Centre [PLC] and Foothills Medical Centre [FMC]). Data were collected between October 2014 and March 2016. The UAH and FMC are comparably large academic tertiary care facilities and the GNCH and PLC are comparable community hospitals. The mean number of emergency department visits annually was 66,003 (UAH), 67,300 (GNCH), 80,466 (FMC), and 79,172 (PLC) during the period of the study.

All physicians providing care in at least one of the 4 study sites were eligible for inclusion in the direct observation and interview portions of the study. All uses of the ANP related to the patients presenting to the emergency department of any study hospital during the defined study period (October 2014 to March 31, 2016) were considered eligible for inclusion in the administrative database record review.

### Emergency Department Information Systems and Tools

The Edmonton Zone and Calgary Zone emergency departments use different mixes of paper and electronic information systems to provide care and manage health information (Table 1). Patient tracking and emergency department lab results in Edmonton Zone are available on a commercial emergency department information system, but all order entry and charting is done on paper. Diagnostic images are available on stand-alone picture archiving and communication system stations that are not integrated with the emergency department information system. Historical health information in Edmonton is usually obtained via the ANP, which is accessed through a Web browser.

During the study period, care documentation in the Calgary Zone emergency departments was provided using a paper chart. Orders were managed using computerized provider order entry entered into Allscripts, which also provided the relevant laboratory, diagnostic imaging, and pharmaceutical information primarily related to Calgary Zone. ANP is available as a parameter-based launch from within the Allscripts interface. As Allscripts in Calgary contains most relevant patient data, it was hypothesized that the use of ANP would be much less in the Calgary zone and primarily related to patient care that occurred outside the Calgary Zone.

### Ethics and Approvals

Research ethics approval was obtained from both the University of Alberta (Approval Pro00033249) and the University of Calgary (Approval REB13-0204) for the observation and interview portions of the study. The database review including the ANP access audit data was obtained following guidelines from provincial legislation that permits the use of data for system quality improvement purposes, which was the primary goal for this data extraction and analysis. The A pRoject Ethics Community Consensus Initiative screening tool was used as a guide in this process, and the resulting risk was assessed as being *low*.

### Structured Clinical Observations

#### Participants

To prepare for the study, emergency department administrators, physicians, nurses, and allied health staff were informed about the study using email notices, visits to departmental meetings, and posted notices in the emergency departments. Physicians were approached by observers during their regularly scheduled shift. Of the 151 physicians approached regarding participation, 146 provided informed consent and 5 declined to participate. At the initial encounter, physicians provided demographic data, including their age, sex, payment model, comfort with computers (Likert scale 0-10), comfort interacting with EMR (Likert scale 0-10), number of years in practice, and medical credentials.

#### Procedure

Observations were conducted following previously published methods and using the Work Observation Method by Activity Timing (WOMBAT) software (WOMBAT 2.0, University of New South Wales) [20,21]. Research assistants were trained in the study protocol, use of the tablet-based WOMBAT data collection tool, and the emergency department patient-tracking system. The 5 research assistants were trained for at least 12 hours before starting observations. Training also consisted of familiarization with the data collection tool and data definitions (Multimedia Appendix 1), then completion of an observation alongside a more experienced observer.

**Table 1:** Overview of functions available using different applications at the different sites.

Information system function	Application			
	Emergency Department Information System^a^	Alberta Netcare Portal^b^	Sunrise Clinical Manager Clinical Information System^c^	Picture Archiving and Communication System^b^
Track patients	Yes	Not in real time	Yes	No
View lab results	Yes	Yes	Yes	No
View diagnostic imaging results	No	Yes (images and reports)	Yes (images and reports)	Yes (images and reports)
Order medications	No	No	Yes	No
Clinical documentation	No	Text reports (eg, consult letters)	No	No
Handover typed note	Yes	No	Yes	No

^a^Edmonton.

^b^Calgary and Edmonton.

^c^Calgary.

One of the authors (MB) was responsible for training research assistants and ensured that research assistants achieved inter-rater agreement scores of at least 90% with respect to the time spent on tasks and numbers of different tasks recorded before completing solo observations. Each research assistant conducted observations across multiple sites.

The research assistants approached emergency department physicians during the earlier hours of their shift, as this was posited to be when physicians would be more actively involved in direct clinical care, rather than the end of the shift when sign-overs and charting were being completed. After obtaining consent, the research assistants observed the physician for 90 min of their clinical shift and recorded demographic and clinical information regarding each patient seen and the time and clinician reason for each clinical access of ANP or other information systems. Time-stamped records including the tasks being completed, the people who were present, and which information tools were in use were recorded for later analysis.

Observations were balanced for the time of day (morning 8 am to 12 pm, afternoon 12 pm to 4 pm, and evening 4 pm to 8 pm) and time of week (midweek, weekend, Monday, and Friday). Qualitative field notes supported the capture of additional contextual information, including the number of learners (medical students and residents) on shift with the physician, the busyness of physician shift, type of patients seen (ie, chronic, complex vs ambulatory, pediatric vs geriatric), and type of presentation, when possible. Inter-rater reliability was calculated during training sessions where learning observers were trained to score the activities alongside more experienced observers, including the second author. Observers conducted independent data collection after consistently scoring 90% or higher agreement on participant time spent on patient care tasks.

### Administrative Data Matching and Review

Administrative data for visits to the 4 sites, including the length of stay, final disposition (inpatient admission or discharge), and discharge diagnosis were obtained for October 2014 to March 2016. These data are routinely recorded as a part of standard AHS operations and for auditing provider accesses to patient records. emergency department encounters were identified from a national system known as the National Ambulatory Care Reporting System (NACRS) [22], and data matching was conducted using the available patient identifiers in both the NACRS and the ANP access audit databases. The number of screens viewed on the day of an emergency department encounter and the subsequent day was retrieved from the access audit data. The 2 datasets were matched using a common Unique Lifetime Identifier, a provincially assigned specific number used for patient identification purposes. Screen views were included in the analysis if they happened on either the day of the emergency department visit or the subsequent day. A screen view was defined by a change in the context of the information that an emergency department physician accessed on the ANP (Multimedia Appendix 2). For example, after searching for and identifying a patient, the first screen is a demographics screen with a Clinical Document Viewer with links to various types of clinical information. Clicking on a link might provide a single lab value, a lab flow sheet with multiple values, a textual report, a diagnostic image, or an electrocardiogram, each on a different screen view within the ANP, and each counting as a separate access.

### Analysis

Quantitative analysis was completed using R (3.4.3, The R-Foundation) and Tableau (10.3). Simple linear regression was completed to evaluate potential effects of demographic, practice, and site-related explanatory variables on the percentage of time spent using ANP and other emergency department information systems tools such as emergency department information system and picture archiving and communication system. Additionally, structured physician observation records included contextual notes to ascertain what information needs were being met when accessing information tools during observations. Differences between sites were evaluated using a Kruskal-Wallis analysis of variance. The initial study questions were used to develop the semistructured questionnaire to evaluate what value clinicians found in the tools they used, including the iEHR, which were evaluated against the quantitative results to support triangulation of the findings.

## Results

### Structured Clinical Observations

**Figure 1:**
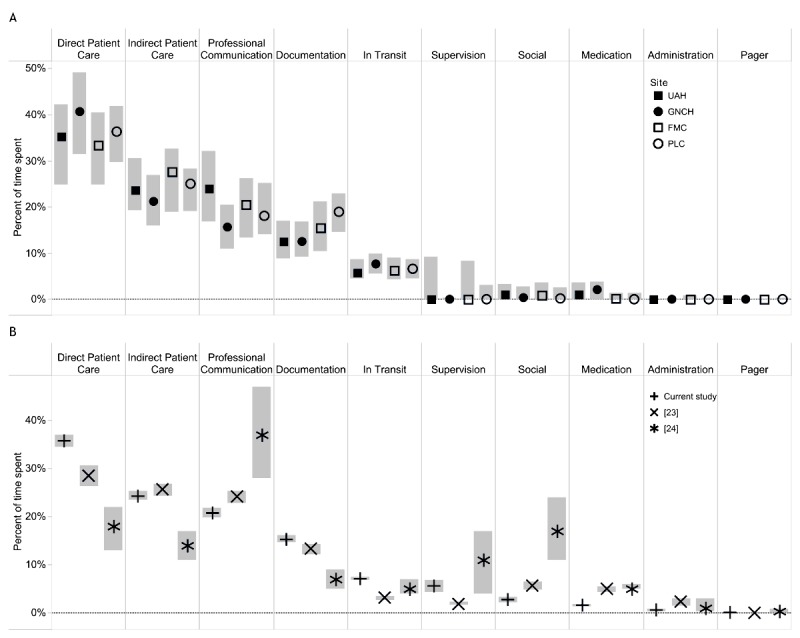
Comparison of proportions of time spent on different tasks in the 4 emergency departments (A) and 2 other studies using the Work Observation Method by Activity Timing (WOMBAT) method (B). (A) The median and interquartile range of the data for individual observations of physician work at 4 EDs are shown by the symbol and the gray bars. The different task categories are labeled on the x-axis at the top of the figure. University of Alberta Hospital: filled squares; Grey Nuns Community Hospital: filled circles; Foothill Medical Centre: empty squares; Peter Lougheed Centre: empty circles. (B) The mean time spent and 95% CIs are reported for this study (+ sign), an Australian study of emergency department physician work (at a registrar training level comparable with the participants in the current work; x sign), and an Australian study of hospital ward physician work (* sign).

To evaluate the context of emergency department physician use of ANP during clinical work, research assistants recorded structured clinical observations at the 4 research emergency departments. Between October 2014 and July 2015, 376 structured, 90-min clinical observations were completed with 142 physician participants (Sites UAH=97, GNCH=94, FMC=99, and PLC=83). As emergency department physicians in Calgary provide care across the different sites in each city, 26 participants were observed at more than one site. For over 566 hours of observation, study personnel used the tablet-based WOMBAT app to record which information resources were being used, including the content types they accessed and the proportion of time emergency department physicians spent accessing the ANP during each 90-min observation. To evaluate whether the method was correctly applied, the percentages of time spent on high-level task categories were calculated and compared for the 4 emergency departments (Figure 1). The proportions of time spent on the different categories were compared and found to be statistically similar across the sites. The mean proportions of time spent (with 95% CIs) on task categories in this study were compared with previously published literature (Figure 1). These percentages of time spent on different tasks while physician participants provided emergency department care in Australian hospital wards were similar to the values reported in this study than the percentages of time spent on the high-level task categories by physicians providing care in Australian hospital wards [20,23].

**Figure 2:**
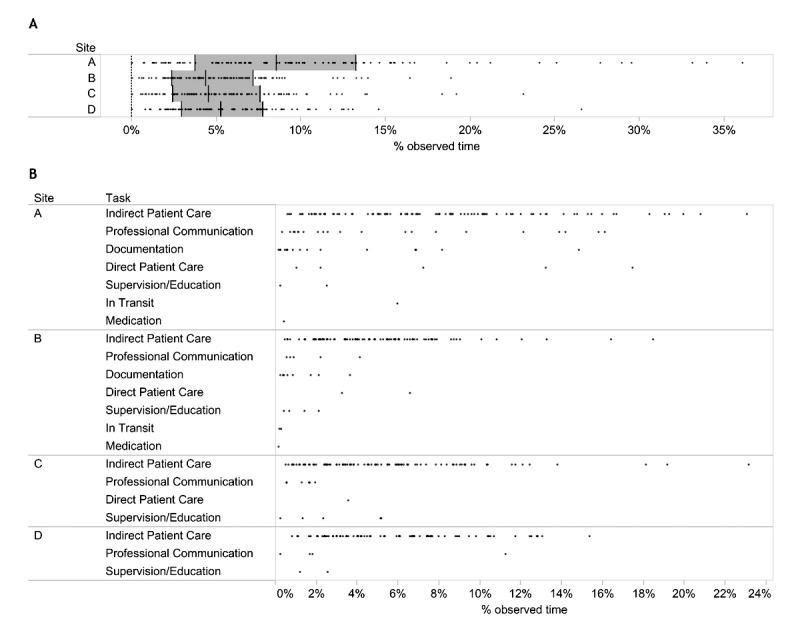
Percentage of observed participant time spent using Alberta Netcare Portal (ANP). (A) Each circle represents the percentage of time spent using ANP for a single 90-min observation session (n=376). The middle black bars represent the median percentage of time spent using ANP and the gray rectangles represent the interquartile range. University of Alberta Hospital site is A, Grey Nuns Community Hospital is B, Foothill Medical Centre is C, and Peter Lougheed Centre is D. (B) Circles represent the proportion of time spent during individual observation sessions using ANP while completing the tasks named at the left-hand side.

The median percentage of physician participant time spent using ANP was 8.5% at UAH (Figure 2; interquartile range, IQR, 3.7%-13.3%), 4.4% at GNCH (IQR 2.4%-4.4%), 4.6% at FMC (IQR 2.4%-7.6%), and 5.1% at PLC (IQR 3.0%-7.7%). The value at UAH was significantly higher than the other sites (*P*<.001). The tasks physicians were observed completing most often while using ANP are identified in Figure 2.

ANP was used during 20.9% of time spent on indirect patient care, including reviewing patient information. ANP was used alongside discussions. Of the total recorded time, 1.5% of the recorded time was spent on Professional Communication. Other tasks that were completed while physicians used ANP included Documentation (0.59%), Direct Patient Care (0.55%), Supervision/Education (0.26%), In Transit (0.06%), and Medication-related tasks (0.01%).

To evaluate other factors that may affect the observed use of ANP, physician demographic and practice characteristics were recorded and simple linear regression was used to identify potential effects on ANP use (Table 2). No statistically significant effects were found.

### Administrative Data Matching and Review

**Figure 3:**
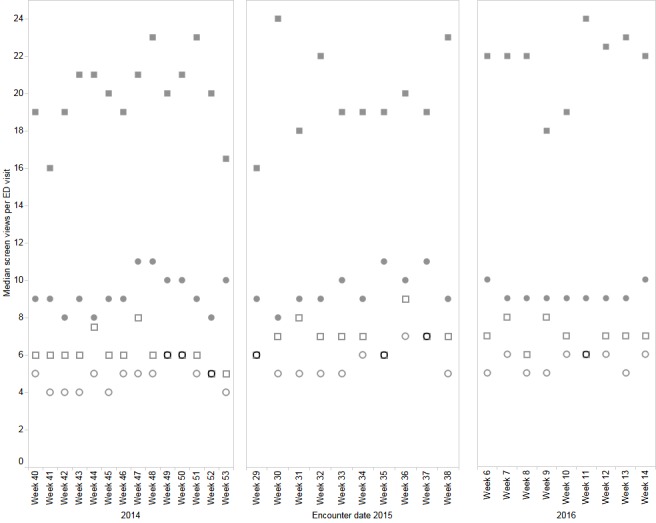
Relative usage of Alberta Netcare Portal across 4 emergency departments from 2014 to 2016 based on access audit data. Each symbol represents the median weekly count of screens accessed per patient visit to the 4 emergency departments. University of Alberta Hospital: filled squares; Grey Nuns Community Hospital: filled circles; Foothill Medical Centre: empty squares; Peter Lougheed Centre: empty circles.

Audit logs from the same time period as the structured clinical observations (October 2014 to March 31, 2016) were compared with the triangulated findings between the 2 methods of evaluating clinician use. Alongside the structured clinical observations, administrative access audit data from ANP were linked with a routinely generated dataset on emergency department visits. Patient visits to the 4 study emergency departments between October 2014 and April 2016 were identified using administrative data and matched with access audit data to derive the number of ANP screens accessed during 3 periods of time (October 2014 through January 2015, July 2015 to September 2015, and February 2016 to April 2016). An ANP access relating to an emergency department visit was defined as the use of the ANP after presentation to the emergency department on the same and next calendar day. During the study period, emergency department physicians at all sites accessed 1,994,334 lab results, 763,334 diagnostic imaging results, and 666,222 textual reports. Over 376 observations and 142 emergency department physicians, the mean and median number of ANP screens viewed per 90-min observation in the Edmonton sites were 53 (UAH) and 27 (GNCH). In the Calgary sites, the mean number of ANP screens viewed was 15 (FMC) and 13 (PLC; Figure 3). Screen accesses were calculated at each site, and a median of 20 screens was accessed per patient visit at UAH (IQR 6-67), 9 at GNCH (IQR 4-29), 7 at FMC (IQR 2-18), and 5 at PLC (IQR 2-14). When compared with the structured clinical observations, the statistical analysis of screen access data also showed that ANP was used more at UAH than the other sites.

**Figure 4:**
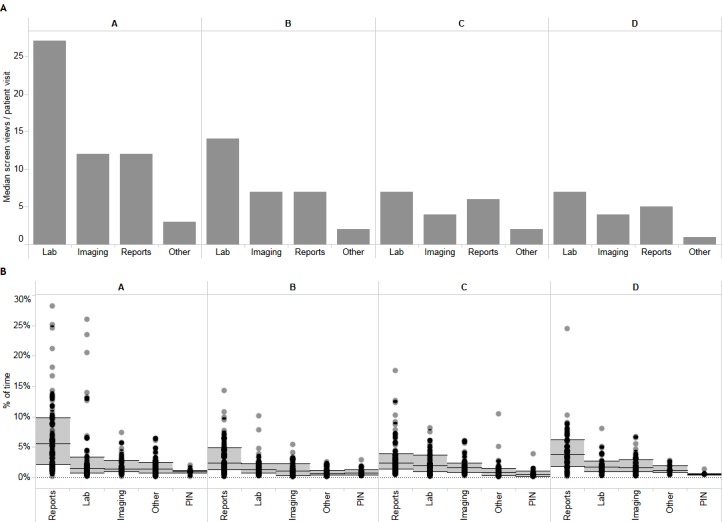
Usage of the different clinical functions in Alberta Netcare Portal based on (A) median counts of screen views from access audit data and (B) observed app usage as measured by the percentage of time spent. The data are shown for each of the 4 sites (A: University of Alberta Hospital; B: Grey Nuns Community Hospital; C: Foothill Medical Centre; D: Peter Lougheed Centre) for laboratory results (Lab), diagnostic imaging (Imaging), textual reports (Reports), Pharmaceutical Information Network (PIN; ie, dispensed medication information), and other information (Other). Circles in (B) represent values for individual 90-min observations. Horizontal black bars and gray areas in (B) represent the median and 95% CIs, respectively.

Among the different content types available in ANP, laboratory and imaging data were accessed more often than transcribed reports, dispensed drug information, or information not categorized elsewhere (eg, electrocardiograms; Figure 4). Physicians were observed spending relatively more time reviewing textual reports (mean 4.6%, 95% CI 4.1-5.1) than lab results (mean 2.6%, 95% CI 2.1-3.1) or diagnostic imaging (mean 1.9%, 95% CI 1.6-2.1; Figure 4).

**Table 2:** Effect of demographic and practice characteristics on Alberta Netcare Portal (ANP) use as measured by the proportion of time spent.

Demographic and practice characteristics		Sites A and B (n)	Sites C and D (n)	Relationship with observed ANP usage (*P* value)
**Age (in years)**				.68
	<30	2	3	—^a^
	30-39	24	38	—
	40-49	23	25	—
	50-59	7	10	—
	>60	6	4	—
**Sex**				.06
	Female	13	17	—
	Male	49	63	—
**Credentials**				.06
	Medical Doctorate	41	78	—
	Fellow of the Royal College of Physicians of Canada–Emergency Medicine	27	44	—
	American Board of Emergency Medicine	4	5	—
	Special Competence in Emergency Medicine	28	29	—
	Other (PhD)	3	0	—
**Payment scheme**				.47
	Fee for service	54	73	—
	Salary	7	9	—
	Mixed-compensation model	4	0	—
Years in practice, mean (SD)		13.3 (10)	12.9 (9)	.29
Comfort with computers^b^, mean (SD)		8.1 (1.6)	8.3 (1.6)	.35
Comfort with electronic medical records^b^, mean (SD)		7.9 (1.6)	8.1 (1.7)	.93

^a^Not applicable.

^b^Likert scale 0-10; 10=completely comfortable.

## Discussion

### Principal Findings

This work triangulates data to evaluate the use of an iEHR (health information exchange-type) in 4 Canadian emergency departments. To our knowledge, there have been no prior Canadian studies describing the use and utility of an iEHR in the emergency department or any other clinical setting. Across Canada, iEHRs are at various stages of implementation and maturity and have evolved according to provincial and territorial strategies and priorities [16]. The Alberta Netcare Portal iEHR is widely used throughout Alberta. A previous study of ANP use showed 76% of users indicated that it helped provide quality patient care, whereas 82% felt that it integrated easily into their clinical workflow [18]. Canadian survey data from 2006 to 2014 demonstrate that in 6 provinces, the Canadian iEHR is perceived to have positive outcomes in terms of user satisfaction, impact on quality of care, and impact on productivity [1,15].

In the emergency department setting in other jurisdictions, these types of read-only iEHRs have been shown to be valued by physicians [2,24,25] and to increase the ability to identify frequent emergency department users [26]. Usage of iEHRs has been reported to vary across different practice settings even when the technical configuration is identical [27], potentially resulting in inconsistent and poorly understood clinical impacts [28].

The triangulation of results in our study, along with the comparison of the time and motion data with results that have been previously reported using the same method in other countries and care environments, supports the findings. Clinicians spent a significant amount of time accessing the various areas of the ANP and reviewing information relevant to patient care, consistent with their content. The access audit data complement these findings. The counts of screen accesses show information searching behaviors that are temporally linked to individual patient emergency department visits and implying they are of important clinical value. Physician perceptions of the ANP were explored in more detail via semistructured interviews and are reported elsewhere.

In Alberta, it is clear that the ANP has become an integral part of emergency department care and is used extensively, with the highest observed usage in the complex environment of a paper-based tertiary care academic center. ANP was more heavily used at the UAH and GNCH (where they document care primarily on paper and order laboratory testing, diagnostic imaging, and other interventions either using paper forms or by verbally or telephonically communicating with different services), compared with sites using a clinical information system that provided similar information but greater integration into point-of-care workflows. In contrast to the UAH, the tertiary care academic site in the Calgary Zone (FMC) had an emergency department clinical information system, and their relative use of ANP was considerably less—most of the information related to their local patients was usually embedded within the context of the emergency department clinical information system.

EHR disuse may result when users and owners do not accrue the benefits of their use [29]. Here, we found that ANP use was independent of the characteristics we evaluated and consistent with emergency department physicians perceiving that content available via ANP supported medical decision making. In particular, information related to the medical management of patients and clinical decision support was not available in ANP and was noted as a potential area of improvement by participants in the structured clinical observations.

Given that the ANP was utilized less frequently in Calgary emergency departments where they were able to access much of the ANP information in their regional clinical information system, as clinical information system implementations increase across the country, the role of separate provincial iEHRs needs further evaluation. Physicians also seek to have clinical documentation, electronic referral, computerized provider order entry, and clinical decision support all accessible in a single point-of-care system to support their clinical practice, rather than logging in and out of multiple systems. Currently, the iEHR is a read-only app (with some small exceptions) and does not support the clinical documentation and ordering needs. Potential important benefits of clinical information system and electronic records relate to patient safety, particularly around medication management and clinical decision support [2,5-7,18], which are not available in the ANP.

### Limitations

Although hybrid paper and computerized practice environments are common in Canada, our results may or may not generalize to other settings that do not have the particular mix of these systems present in the study emergency departments. The Alberta context, where an iEHR is available alongside a more transactional clinical information system in some sites, afforded a unique opportunity to study its use and clinical utility systematically.

In defining the access of ANP related to an emergency department visit, the audit logs were not detailed enough to identify from what part of the hospital the ANP was accessed. Our ANP accesses may be slight overestimates when a patient was admitted to the hospital and the ANP was accessed by the inpatient care team. We believe this possibility to be small because the majority of patients presenting were not admitted to an inpatient service, and it can take many hours for an admitted patient to move from an emergency department to the inpatient bed.

More generally, partially adopted clinical documentation applications may lead to hybrid environments where some forms of information are charted on paper and others are charted in a clinical information system [30-32]. This work illuminates the need to use multiple methods while evaluating the impacts of different methods of information storage and retrieval in the context of fast-paced emergency department care while showing the clinical utility of a single iEHR accessible across a province.

### Conclusions

The current evaluation shows that ANP iEHR is well utilized at the 4 sites studied, and physicians participating in the study perceived ANP has a positive impact on knowledge of their patients, patient safety, and quality and continuity of care. Physicians at the paper-based tertiary care hospital utilized ANP markedly more than those at the clinical information system-based tertiary care hospital or the 2 community hospital sites. Physicians working at all 4 sites accessed lab results and diagnostic imaging more often than textual reports, such as discharge summaries and operative reports, but spent relatively more time reviewing textual reports. Physician demographic and practice characteristics did not predict this common usage pattern. In its current form, ANP features that could be enhanced include electronic referral, clinical documentation, and medication ordering and management. Given the trend of moving toward comprehensive clinical information systems to run hospital systems, the future design, development, and importance of the iEHR need further evaluation.

## Appendix

Multimedia Appendix 1 WOMBAT Workflow Definitions.

Multimedia Appendix 2 Access audit data definitions.

## Abbreviations

AHS: Alberta Health Services
ANP: Alberta Netcare Portal
EHR: electronic health record
FMC: Foothill Medical Centre
GNCH: Grey Nuns Community Hospital
iEHR: interoperable electronic health record
IQR: interquartile range
NACRS: National Ambulatory Care Reporting System
PLC: Peter Lougheed Centre
UAH: University of Alberta Hospital
WOMBAT: Work Observation Method by Activity Timing
