# Supplementary material for: Emergency Physician Use of the Alberta Netcare Portal, a Province-Wide Interoperable Electronic Health Record: Multi-Method Observational Study
Source: JMIR Med Inform. 2018 Sep 25;6(3):e10184. doi: 10.2196/10184 (PMC6231720; doi:10.2196/10184)
Supplement: Multimedia Appendix 1 [file medinform_v6i3e10184_app1.pdf]

Definitions
Work Measurement Tool

Physician

Version Date: 2014-Sept-16

Summary of Observation Tool:

Task

Direct Care	Administrative
Indirect Care	In Transit
Reviewing Information	Supervision/Education
Medication	Social
Documentation	Pager
Professional Communication	

Who

Patient	Relative
Nurse	Allied Health
Doctor	Other – See Note
Pharmacist	Alone/No One

Tool (Optional)

Permanent Paper Record	Phone
Paper	Phone Call
CIS/SCM/EDIS	Vital Signs
Alberta Netcare Portal	Medication pumps
Personal Computer - Desk	Other – See Note
Tablet	

Alberta Netcare Portal

Laboratory Results	Pharmaceutical Information Network/PIN
Imaging	Reports
Other – See Note	

Desk-PC

PACS	UptoDate
eCPG	Google – Other Internet
Other – See Note	

Section 1 – Work Task Categories

Direct Patient Care

Any activity directly related to patient care.

Includes
- Admitting a patient
- Examining/reviewing a patient
- Performing medical procedures
- Assisting other staff with procedure
- Escorting a patient
- Communicating with patient/relative
- Taking a history

Excludes
- medication related activities
- documenting
- reviewing documentation/results
- planning care
- communicating with staff member

Note:
- All communication with patient/relative is defined as direct patient care
- When the participant is discussing medications with a patient (eg. as part of history taking and review), this is defined as a medication related event.

Indirect Patient Care

Any activity indirectly related to patient care.

Includes
- washing hands
- gathering & returning equipment
- cleaning up after a procedure
- watching monitors

Excludes
- medication related activities
- documenting in patient notes
- communicating with staff member
- communicating with patient/relative
- retrieving information (from temporary or perm record, or computer)
- reading & reviewing documents
- checking results

Note:
Monitors, ventilators, and other electronic patient care equipment should be coded as ‘computer’.

Retrieving Information

Any activity related to reviewing and retrieving information in one or more information resources.

Sub category: logging in

- entering credentials to access Netcare, EDIS, SCM

Includes:

- retrieving information (from temporary or perm record, or computer)
- reading & reviewing documents
- checking results

Excludes:

- Documentation tasks including writing in patient paper or electronic charts.
- Writing orders

Medication

Any activity that relates to medication for a particular patient

Includes:

Finding orders: looking for medication charts/medical records with drug

Prescribe drug: writing up a new order, changing orders, requesting a verbal order, writing discharge scripts, obtaining drug authority numbers

Discuss: Talking about a drug with another health professional &/or patient/relative

- choice of drug &/or dosage
- side effects
- discharge education
- efficacy
- administration protocols

Review: Looking over drug orders as part of planning care

Excludes:

looking for notes in general

Documentation

Any recording of patient information on paper or computer

Includes
- writing on temporary record (eg own list)
- writing in patients' notes
- getting physicians to sign-off on non-medication orders
- discharge summaries

Excludes
- medication chart documentation

Professional Communication (Prof Comm)

Any work-related discussion with another staff member

Includes
- requesting medical or nursing consult or review
- planning care with any health professional
- handover/parts of a ward round

Excludes
- medication related discussion
- communication with pt or rel

Administrative

Any administrative activity that is not related to direct or indirect individual patient care. Includes activities that relate to the running of the unit in general (but aren't related to direct or indirect patient care)

Includes
- duty rosters
- employment issues
- bed allocations
- coordination of staff activities
- staff meetings (not case or clinical meetings
- unit related
- unit orders for imprest stock

Excludes
- handover
- rounds

In Transit

Work related movement between patients and between tasks

Includes
- movement when the participant exits a patient room

Excludes
- movement between patients in a shared room
- movement within a single room

When the participant arrives at another task or patient, "In Transit" ceases and the next appropriate category is chosen for that active task.

eg. participant leaves room to get equipment = in transit, participant returns with equipment = indirect care

Supervision/Education

Active supervision or teaching of another staff member or student
Note: When the participant is actively supervising, "supervision" is selected and all tasks normally undertaken by the participant are added under "multitasking"
Attending education sessions eg. grand rounds

Social

Any social or personal activity or discussion. Includes personal phone calls, tea & personal breaks

Includes	Excludes
- personal phones calls, tea, & personal breaks<br>- bathroom breaks<br>- reading books/magazines	

Pager

Whenever the participant's pager alerts, pager is to be entered as an interruption.

Includes	Excludes
- reading pager<br>- returning call	- calling / having paged other healthcare providers See Prof Comm
